# Impact of Neoantigen Expression and T-Cell Activation on Breast Cancer Survival

**DOI:** 10.3390/cancers13122879

**Published:** 2021-06-09

**Authors:** Wenjing Li, Amei Amei, Francis Bui, Saba Norouzifar, Lingeng Lu, Zuoheng Wang

**Affiliations:** 1 Department of Mathematical Sciences, University of Nevada, Las Vegas, NV 89154, USA; liw12@unlv.nevada.edu; 2 School of Life Sciences, University of Nevada, Las Vegas, NV 89154, USA; buif1@unlv.nevada.edu (F.B.); norous1@unlv.nevada.edu (S.N.); 3 Department of Chronic Disease Epidemiology, Yale School of Public Health, New Haven, CT 06520, USA; lingeng.lu@yale.edu; 4 Department of Biostatistics, Yale School of Public Health, New Haven, CT 06520, USA

**Keywords:** breast cancer, neoantigen expression, T-cell activation status, DNA repair gene, overall survival

## Abstract

**Simple Summary:**

Neoantigens are novel proteins presented on the cell surface and derived from the accumulation of somatic mutations in tumor cells. They can be recognized by the immune system and may play a crucial role in boosting immune responses against tumor cells. The impact of neoantigen expression and T-cell activation status on overall survival was investigated in a breast cancer cohort. We found that high neoantigen expression and T-cell activation status was correlated with improved patient survival in the study population. This result supports that neoantigens are promising to serve as immunogenic agents for immunotherapy in breast cancer.

**Abstract:**

Neoantigens are derived from tumor-specific somatic mutations. Neoantigen-based synthesized peptides have been under clinical investigation to boost cancer immunotherapy efficacy. The promising results prompt us to further elucidate the effect of neoantigen expression on patient survival in breast cancer. We applied Kaplan–Meier survival and multivariable Cox regression models to evaluate the effect of neoantigen expression and its interaction with T-cell activation on overall survival in a cohort of 729 breast cancer patients. Pearson’s chi-squared tests were used to assess the relationships between neoantigen expression and clinical pathological variables. Spearman correlation analysis was conducted to identify correlations between neoantigen expression, mutation load, and DNA repair gene expression. *ERCC*1, *XPA*, and *XPC* were negatively associated with neoantigen expression, while *BLM*, *BRCA*2, *MSH*2, *XRCC*2, *RAD*51*, CHEK*1, and *CHEK*2 were positively associated with neoantigen expression. Based on the multivariable Cox proportional hazard model, patients with a high level of neoantigen expression and activated T-cell status showed improved overall survival. Similarly, in the T-cell exhaustion and progesterone receptor (PR) positive subgroups, patients with a high level of neoantigen expression showed prolonged survival. In contrast, there was no significant difference in the T-cell activation and PR negative subgroups. In conclusion, neoantigens may serve as immunogenic agents for immunotherapy in breast cancer.

## 1. Introduction

DNA damage and genetic mutations accumulate during cancer development [1,2]. Somatic mutations in tumor cells may function as neoantigens which consequently elicit immune response against the tumor. Neoantigens are often recognized as foreign by adaptive immune cells [3] and demonstrate high immunogenicity [4]. The host immune system’s inbuilt ability to distinguish foreign proteins can induce the immune response and destruct malignant T-cells [3,5]. Neoantigens are processed and presented by major histocompatibility complex (MHC) molecules, which are then recognized by T-cell receptors [6,7]. CD4^+^ and CD8^+^ T-cells are subsequently activated to evoke cytotoxic T lymphocyte responses to eliminate tumor cells [6,7]. As ideal immunotherapeutic targets, neoantigens have shown great promise in various human malignancies and significantly reduce autoimmunity risk with minimally assured immune tolerance [8]. Therefore, patient-specific neoantigen is presumed to enhance the efficacy of cancer immunotherapy and serve as a beneficial predictor of cancer prognosis in clinical trials on immunotherapeutic checkpoint inhibitors or vaccines [9].

CD8^+^ T-cell exhaustion and enhanced regulatory T-cell function are important in immune editing and escape [10]. Effector CD8^+^ T-cell activation, one of the critical components in the anti-tumor immunity, has been shown to be positively associated with neoantigens and impact patient survival [10,11]. Immunotherapy may fail for multiple reasons including lack of T-cells that are capable to recognize neoantigens, suppression of such T-cells [9], and insufficient amount of expressed neoantigens. A series of inhibitory molecules are allied to effector CD8^+^ T-cell exhaustion, causing tumor cells to escape from immune surveillance [11,12]. Immune checkpoints, such as cytotoxic T lymphocyte-associated antigen 4 (CTLA-4) and programmed cell death protein 1 (PD-1), transmit negative signals to CD8^+^ T-cell and reduce tumor-infiltrating lymphocytes during the anti-tumor process [13,14,15]. Patients with high T-cell activation scores have prolonged survival in breast cancer [10]. Deficient DNA repair yields genomic instability and promotes clinical responses to immune checkpoint inhibitors (ICIs). Application of ICIs has been approved to treat the subgroup of patients with cancer resulting from genomic instability. Several DNA repair pathways have been found to confer more neoantigens [16]. Therefore, investigating the relationship between neoantigens and DNA repair pathways is likely to provide new insights in immunotherapy.

Studies have proposed association of neoantigen with survival in cancer patients [17,18]. There exists positive correlation between predicted neoantigen load and breast cancer survival [6]. In this study, we aim to study whether neoantigen expression, together with T-cell activation status, affects patient survival in breast cancer. We also explored the relationship between neoantigen expression and DNA repair genes in breast cancer. To our knowledge, this is the first study to illuminate that neoantigen expression is positively associated with improved breast cancer survival.

## 2. Materials and Methods

### 2.1. Study Subjects and Data Sources

This study included 1081 female patients diagnosed with primary breast cancer. Clinical data on the patients were obtained from The Cancer Genome Atlas (TCGA) breast invasive carcinoma study (http://www.cbioportal.org/, assessed on 1 July 2018) The somatic mutation data on 90,969 mutant sites were downloaded from Genomic Data Commons (GDC) data portal (https://portal.gdc.cancer.gov/; assessed on 26 September 2019). The normalized RNA sequencing data which provide gene expression levels of 60,483 mRNA transcripts were downloaded from GDC data portal. All data were downloaded using an R package ‘TCGAbiolink’ [19]. The binding affinities between mutant peptides and HLA alleles, as well as the affinities with the corresponding wild-type peptides, were obtained from a previous study in which the binding affinities were reported using two measures, half maximal inhibitory concentration (${\mathrm{IC}}_{50}$) value and percentile rank score [20]. The ${\mathrm{IC}}_{50}$ value reports a direct binding affinity prediction, and the percentile rank score reports relative binding affinity with a specific HLA allele compared to a random peptide. Mutant peptide with ${\mathrm{IC}}_{50}$ < 500 nM or percentile rank score ≤ 2 is commonly considered as a potential neoantigen, indicating a strong bind to HLA [20,21,22,23]. After merging all data together, clinical information, somatic mutations, mRNA expression, and peptide binding affinities of 729 patients were available for further analysis.

### 2.2. Statistical Analysis

Neoantigens were predicted based on peptide-HLA binding affinities. A mutant peptide is considered as a neoantigen if (1) the predicted binding affinity with the patient’s HLA alleles satisfies that ${\mathrm{IC}}_{50}$ < 500 nM or percentile rank score ≤ 2, and (2) the binding affinity of the corresponding wild-type peptide satisfies that ${\mathrm{IC}}_{50}$ ≥ 500 nM or percentile rank score > 2 [22]. Several pathogenic variants—such as *BRCA*1*/*2, *KRAS*, *NRAS*, *PTEN*, *TP*53, and *PALB*2—tend to increase breast cancer risk. Individuals who carry the susceptibility alleles at these variants are more likely to develop breast cancer. In this study, we excluded these pathogenic genes for neoantigen prediction to reduce false-positive causality [24]. For each mutant peptide, neoantigen expression was the expression level of the gene where the mutant resides if it was predicted as a neoantigen. An individual’s neoantigen expression was the total gene expression levels across all predicted neoantigens.

Pearson’s chi-squared tests were used to assess the associations between neoantigen expression and clinical variables including estrogen receptor (ER) status, progesterone receptor (PR) status, human epidermal growth factor receptor 2 (HER2) status, molecular subtype, disease stage, and histological type. Binary neoantigen expression level, high or low, was defined based on the cutoff value of neoantigen expression determined by an algorithm to maximally separate the survival curves between the two groups using an R package ‘survminer’. Spearman correlation analysis was conducted to evaluate the correlations between neoantigen expression, mutation load, and the expression levels of 24 DNA repair genes, *APEX*1, *ATM*, *BLM*, *BRCA*1, *BRCA*2, *ERCC*1, *ERCC*4, *ERCC*6, *FANCG*, *MLH*1, *MSH*2, *MSH*3, *MUTYH*, *OGG*1, *RPAP*1, *XPA*, *XPC*, *XRCC*1, *XRCC*2, *XRCC*3, *XRCC*4, *RAD*51, *CHEK*1, and *CHEK*2 [7,25,26,27,28]. Bonferroni adjustment was used to correct for multiple hypothesis testing.

A weighted T-cell activation score was calculated for each patient based on 13 genes that are related to T-cell activation status as described previously [10]. We further divided patients into two groups, activation and exhaustion. Multivariable Cox proportional hazard models were used to evaluate the relationship between neoantigen expression and overall survival. Patient’s age at diagnosis, ER status, disease stage, histology, and T-cell activation status were included as covariates in the models to obtain adjusted hazard ratios (HRs) with 95% confidence intervals (CIs). The effect of neoantigen expression on overall survival was further assessed in each subgroup stratified by the T-cell activation status, ER status, and PR status, respectively. In all statistical analyses, results were considered significant when *p*-values were less than 0.05. All analyses were performed in R (https://www.r-project.org/; version 3.5.1, updated on 1 March 2019)

## 3. Results

### 3.1. Clinical and Pathologic Characteristics of Patients

Characteristics of the 729 breast cancer patients are displayed in Table 1. The average age at diagnosis was 57.7 (range 26–90) years old. Among the 709 patients with disease stage information, 17.6% (*n* = 125) were diagnosed at stage I, 57.6% (*n* = 408) were at stage II, and 24.8% (*n* = 176) were diagnosed at an advanced stage (III or IV). Among the 691 patients with a known ER status, 77.1% (*n* = 533) were ER-positive. Among the 689 patients with a known PR status, 68.4% (*n* = 471) were PR-positive. Among the 498 patients with a known HER2 status, 23.5% (*n* = 117) were HER2-positive. All patients had malignant breast cancer with the predominant histological type of ductal carcinoma (83.0%, *n* = 604), followed by lobular (8.5%, *n* = 62), mix (6.0%, *n* = 44), and other (2.5%, *n* = 18). 495 patients had molecular subtype information available: 79.4% (*n* = 393) luminal, 14.3% (*n* = 71) basal-like, and 6.3% (*n* = 31) HER2-enriched. The average length of follow-up in the 729 patients was 42.8 (range: 0–282.7) months and 112 patients died during the follow-up period.

### 3.2. Correlation between Neoantigen Expression and Clinical Pathological Variables

Neoantigen expression was calculated using the expression values of all predicted neoantigens for each patient. The median of neoantigen expression was 57.38 (range: 0–7038.03). A cutoff value of 16.10 was chosen to classify the patients into two groups: 569 patients (78.1%) in the high neoantigen expression group, and 160 patients (21.9%) in the low neoantigen expression group. Correlations between neoantigen expression and clinical pathological variables—including the ER status, PR status, HER2 status, molecular subtype, disease stage, and histological type—were evaluated (Table S1). Neoantigen expression was significantly associated with the ER status (*p* = 0.016), PR status (*p* = 0.006), molecular subtype (*p* = 0.004), and disease stage (*p* = 0.016). There was a borderline significant association between neoantigen expression and the HER2 status (*p* = 0.073). Since ER- tumors are more immunogenic compared with ER+ tumors [29], we found that the proportion of patients with high neoantigen expression in the ER- group is significantly higher than that in the ER+/PR+ group (85.4% vs. 75.3%, *p* = 0.011).

### 3.3. Correlation between Neoantigen Expression, Mutation Load, and DNA Repair Genes

**Figure 1:**
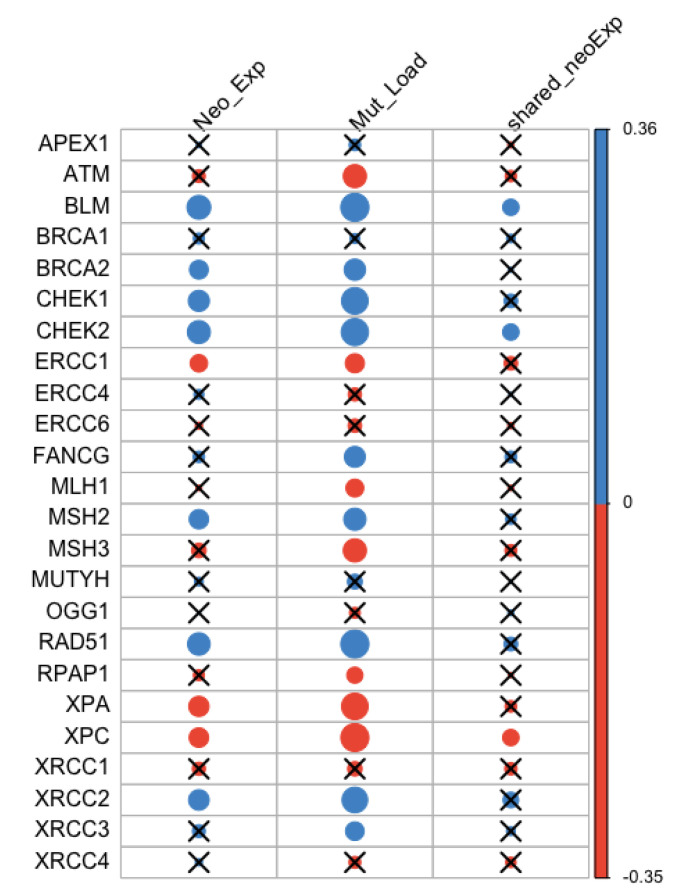
Correlation between neoantigen expression, mutation load, the expression of the most shared neoantigens and DNA repair gene expression. Blue and orange represent positive and negative correlation, respectively. The size of the dot is proportional to the magnitude of the correlation. Insignificant associations were marked with black crosses at the significance level of 0.05.

Correlations between the expression levels of 24 DNA repair genes and neoantigen expression, mutation load, as well as the expression on the most shared neoantigens were assessed (Table S2). The most shared neoantigens were defined as genes on which at least five patients had predicted neoantigens. P-values were adjusted by Bonferroni correction for multiple testing (Figure 1). We identified three negatively correlated genes with neoantigen expression: *ERCC*1 (*r* = −0.14, *p* = $4.95\times 10^{-3}$), *XPA* (*r* = −0.18, *p* = $1.19\times 10^{-5}$), and *XPC* (*r* = −0.17, *p* = $7.58\times 10^{-5}$). They were also negatively correlated with mutation load: *ERCC*1 (*r* = −0.16, *p* = $1.99\times 10^{-4}$), *XPA* (*r* = −0.32, *p* < $5.28\times 10^{-15}$), and *XPC* (*r* = −0.35, *p* < $5.28\times 10^{-15}$). Among them, *XPC* was negatively correlated with the expression of the most shared neoantigens (*r* = −0.12, *p* = 0.025). There were seven DNA repair genes positively correlated with neoantigen expression: *BLM* (*r* = 0.26, *p* = $5.02\times 10^{-11}$), *BRCA*2 (*r* = 0.16, *p* = $3.45\times 10^{-4}$), *MSH*2 (*r* = 0.17, *p* = $9.83\times 10^{-5}$), *XRCC*2 (*r* = 0.19, *p* = $5.18\times 10^{-6}$), *RAD*51 (*r* = 0.23, *p* = $9.46\times 10^{-9}$), *CHEK*1 (*r* = 0.20, *p* = $9.88\times 10^{-7}$), and *CHEK*2 (*r* = 0.24, *p* = $1.68\times 10^{-9}$). They were also positively correlated with mutation load: *BLM* (*r* = 0.36, *p* < $5.28\times 10^{-15}$), *BRCA*2 (*r* = 0.21, *p* = $4.98\times 10^{-7}$), *MSH*2 (*r* = 0.22, *p* = $2.79\times 10^{-8}$), *XRCC*2 (*r* = 0.30, *p* = $5.33\times 10^{-15}$), *RAD*51 (*r* = 0.36, *p* < $5.28\times 10^{-15}$), *CHEK*1 (*r* = 0.32, *p* < $5.28\times 10^{-15}$), and *CHEK*2 (*r* = 0.33, *p* < $5.28\times 10^{-15}$). Among them, *BLM* (*r* = 0.12, *p* = 0.026) and *CHEK*2 (*r* = 0.12, *p* = 0.024) were positively correlated with the expression of the most shared neoantigens.

### 3.4. Association of Neoantigen Expression with Patient Survival

**Figure 2:**
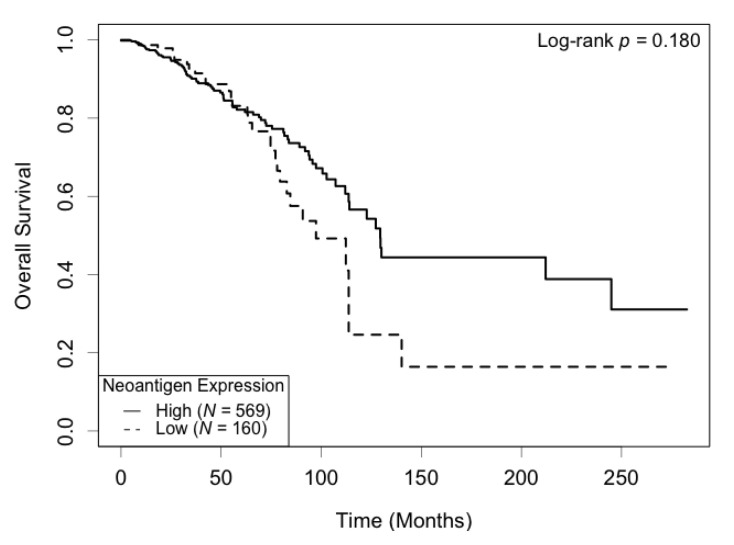
Kaplan–Meier survival curve of neoantigen expression. There was no significant difference in overall survival between patients in high and low levels of neoantigen expression (*p* = 0.180).

**Figure 3:**
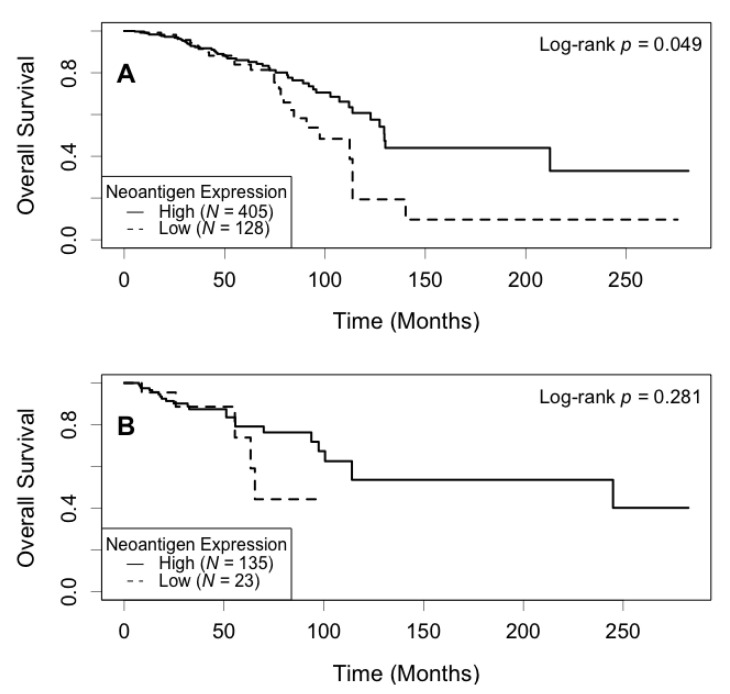
Kaplan–Meier survival curve of neoantigen expression stratified by ER status. (**A**) In the ER positive subgroup, patients with a high level of neoantigen expression had better overall survival compared to those with a low level (*p* = 0.049); (**B**) In the ER negative subgroup, there was no significant difference in survival between patients in the high and low levels of neoantigen expression (*p* = 0.281).

**Figure 4:**
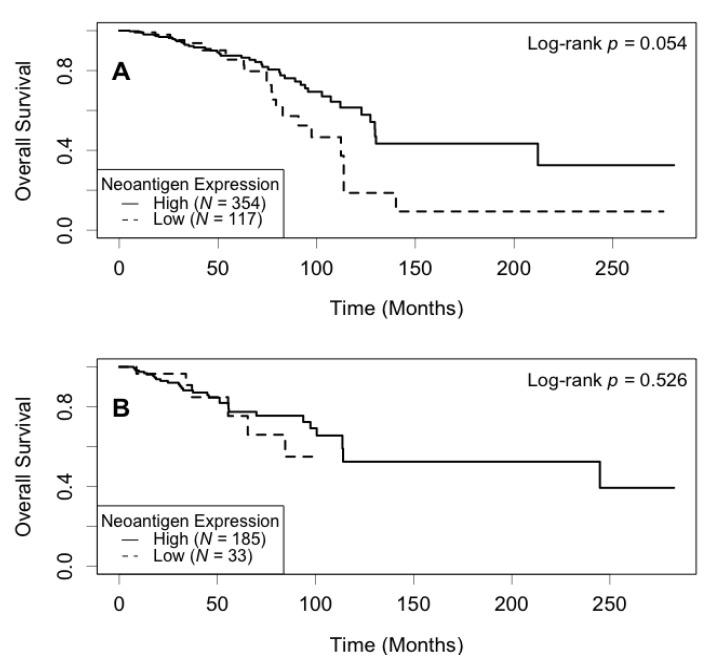
Kaplan–Meier survival curve of neoantigen expression stratified by PR status. (**A**) In the PR positive subgroup, patients with a high level of neoantigen expression had borderline better overall survival compared to those with a low level (*p* = 0.054); (**B**) In the PR negative subgroup, there was no significant difference between patients in the high and low levels of neoantigen expression (*p* = 0.526).

Correlation between neoantigen expression and overall survival was evaluated using Kaplan–Meier (KM) survival curves. The median survival was 129.5 (95% CI: 113.7−∞) months for the high neoantigen expression group, and 97.4 (95% CI: 82.8−∞) months for the low neoantigen expression group (Figure 2). There was a difference of 32.1 months in median survival between the two groups. The log-rank *p*-value was not significant (*p* = 0.180) when comparing the survival curves of the two groups. In the ER-positive subgroup, patients with a high neoantigen expression level had better overall survival compared to those with a low neoantigen expression level (*p* = 0.049, Figure 3A), while in the ER-negative subgroup, there was no significant difference in overall survival between the high and low neoantigen expression groups (*p* = 0.281, Figure 3B). In the PR-positive subgroup, there was a borderline significant positive association between neoantigen expression and overall survival (*p* = 0.054, Figure 4A), while in the PR-negative subgroup, there was no significant difference in overall survival between the high and low neoantigen expression groups (*p* = 0.526, Figure 4B).

The effect of neoantigen expression on overall survival was also assessed using the multivariable Cox regression model, adjusted for patient’s age at diagnosis, ER status, disease stage, histology, and T-cell activation status (Table 2). A higher neoantigen expression level was significantly associated with decreased risk of mortality. The adjusted HR was 0.61 (95% CI: 0.38–0.97, *p* = 0.038) for the high vs. low neoantigen expression group. Overall, patients with a high T-cell activation score (activation, *n* = 184) had better overall survival compared to those with a low T-cell activation score (exhaustion, *n* = 545). The adjusted HR was 0.48 (95% CI: 0.24–0.96, *p* = 0.038) for the activation group vs. the exhaustion group.

We further investigated the relationship between survival and neoantigen expression stratified by T-cell activation status (Table 3). In the exhaustion group, patients with a high neoantigen expression level showed better overall survival compared to those with a low neoantigen expression level. The adjusted HR was 0.55 (95% CI: 0.34–0.89, *p* = 0.016) for the high vs. low neoantigen expression group. In contrast, among patients in the activation group, there was no significant difference in overall survival between the high and low neoantigen expression groups. The adjusted HR was 0.76 (95% CI: 0.08–7.44, *p* = 0.816) for the high vs. low neoantigen expression group.

We also performed analysis stratified by the ER and PR status. In the ER-positive subgroup, there was a borderline significant positive association between neoantigen expression and overall survival (Table 4). The adjusted HR was 0.61 (95% CI: 0.36–1.04, *p* = 0.067) for the high vs. low neoantigen expression group. In the ER-negative subgroup, there was no significant difference in overall survival between the high and low neoantigen expression groups. The adjusted HR was 0.76 (95% CI: 0.26–2.16, *p* = 0.601) for the high vs. low neoantigen expression group. In the PR-positive subgroup, patients with a high neoantigen expression level showed better overall survival compared to those with a low neoantigen expression level (Table 5). The adjusted HR was 0.57 (95% CI: 0.32–0.99, *p* = 0.046) for the high vs. low neoantigen expression group. In contrast, among PR-negative patients, there was no significant difference in overall survival between the high and low neoantigen expression groups. The adjusted HR was 0.67 (95% CI: 0.24–1.84, *p* = 0.439) for the high vs. low neoantigen expression group.

### 3.5. Association of Expression of the Most Shared Neoantigens with Patient Survival

There were 47 genes that had shared neoantigens by patients ranging from 5–23. For example, 23 (3.16%) patients had neoantigens on *MUC*16, also known as cancer antigen 125 (*CA*125), 21 (2.88%) on *PCDHGC*5, 19 (2.61%) on *PCDHAC*2, 12 (1.65%) on *USH*2*A*, 11 (1.51%) on *RYR*3, and 8 (1.10%) on *MUC*17. Correlation between the expression of the most shared neoantigens and overall survival was evaluated using KM survival curves. In the entire study population, patients with a high neoantigen expression level on these genes had better overall survival compared to those with a low neoantigen expression level (log-rank *p* = 0.026, Figure S1). When stratified by T-cell activation status, ER status, and PR status, patients with a high expression level on the most shared neoantigens showed improved overall survival in the T-cell exhaustion group (log-rank *p* = 0.034, Figure S2A), ER-positive group (log-rank *p* = 0.049, Figure S3A), and PR-negative group (log-rank *p* = 0.016, Figure S4B).

The impact of the expression of the most shared neoantigens on overall survival was also assessed using the multivariable Cox regression model, adjusted for patient’s age at diagnosis, ER status, disease stage, histology, and T-cell activation status (Tables S3–S5). High neoantigen expression was significantly associated with decreased risk of mortality in the entire sample (adjusted HR = 0.30, 95% CI: 0.11–0.82, *p* = 0.019) and in the ER-positive group (adjusted HR = 0.28, 95% CI: 0.09–0.90, *p* = 0.033).

## 4. Discussion

Neoantigens were found to be a prognostic factor for overall survival of patients with ovarian cancer [30] and melanoma [31]. Neoantigen vaccines have shown encouraging responses to immunotherapy in clinical trials on melanoma [32] and glioblastoma [33]. Predicted neoantigen load had better prognostic potential than tumor mutation load in the TCGA breast cancer cohort [6]. In this study, we investigated a cohort of 729 patients with breast cancer in TCGA to assess the relationships between neoantigen expression and clinical pathological variables, DNA repair genes, and patient survival.

Immune modulation showed limited efficacy among hormone receptor positive breast cancer patients [34]. In this study, we found that the proportion of patients with high neoantigen expression in the ER-positive group is lower than that in the ER-negative group (76.0% vs. 85.4%, *p* = 0.016). Similar pattern was observed when comparing the PR-positive and PR-negative groups (75.2% vs. 84.9%, *p* = 0.006). Among the three molecular subtypes, the proportion of patients with high neoantigen expression was 77.4% for Luminal, 90.1% for Basal-like, and 93.5% for HER2-enrich subtype. Our results suggest that neoantigen expression varied across hormone receptor status and molecular subtype of breast cancer.

Damage to DNA gives rise to potentially harmful mutations in the genome and blockage of transcription for cell cycle arrest and checkpoints. DNA repair systems are essential for the maintenance of genome integrity. Although defects in DNA repair lead to large amount of mutations, high mutation load results in high neoantigen load [35] and hence high neoantigen expression may lead to greater immunogenicity. We identified three DNA repair genes, *ERCC*1, *XPA*, and *XPC*, that were negatively correlated with tumor mutation load and neoantigen expression, suggesting that dysregulation of DNA repair pathways may promote genome instability and increase the accumulation of DNA lesions and mutations in tumorigenesis [36]. *XPC* was negatively associated with the expression of the most shared neoantigens. *ERCC*1 and *XPA* increase the risk of breast cancer [37,38]. *XPC* polymorphisms are associated with higher susceptibility of breast cancer during the nucleotide excision repair (NER) process [39]. We also found seven DNA repair genes—*BLM*, *BRCA*2, *MSH*2, *XRCC*2, *RAD*51, *CHEK*1, and *CHEK*2—that were positively associated with tumor mutation load and neoantigen expression. Among them, *BLM* and *CHEK*2 were positively correlated with the expression of the most shared neoantigens. Germline mutations in *BRCA*1 and *BRCA*2 account for around 25% of familial breast cancer clustering [40,41,42] and 5–10% risk of all breast cancer cases [43]. *MSH*2 loss may result in advanced breast cancer and its mutations are involved in the development of early-onset breast cancer in the Lynch syndrome family [37,44,45]. Single nucleotide polymorphisms on *XRCC*2 influence breast cancer risk and survival [46]. Pathogenetic mutations or variants in *CHECK*2 and *RAD*51 have been reported to increase the risk of breast cancer [47]. *RAD*51 binds DNA at the damage site for homologous recombination repair. Overexpression of *RAD*51 leads to increased homologous recombination and promotes genomic instability with an increased prevalence of mutations [48]. In breast cancer, *RAD*51 is overexpressed due to excessive transcription and reduced methylation of the gene. The mutation or loss of the tumor suppressor gene *p*53 also contributes to high *RAD*51 expression [48,49]. *CHEK*1 encodes a protein kinase that coordinates the DNA damage response and cell cycle checkpoint response. Overexpression of *CHEK*1 activates the cell cycle and MAPK signaling pathways which were reported to be related to breast cancer onset and development. The activation of the MAPK pathway also plays a role in cell proliferation, cell growth, and breast cancer migration [50,51,52]. *CHEK*2 is another checkpoint gene responsible for regulating cell cycle in the presence of DNA damage. Mutations in this gene prevent the activation of the tumor suppressor gene *p*53, leading to an accumulation of mutations in the genome and the proliferation of tumor cells [53]. Overexpression of *BLM* mRNA was associated with poor breast cancer-specific survival and *BLM* protein also influenced survival, suggesting that *BLM* is a promising biomarker in breast cancer [54].

Our results demonstrated that high neoantigen expression and T-cell activation is associated with decreased mortality risk. The prognostic effect of T-cell activation status in breast cancer was reported in a previous study [10]. Our study was consistent with the finding that activated T-cell status is associated with improved overall survival in breast cancer patients. CD8^+^ cytotoxic T-cells recognize the neoantigens of peptide-MHC class I molecule complexes that were presented on the cell surface and promote patient survival. Our findings confirmed that neoantigens facilitated the anti-tumor immune response and improved the overall outcome. In the T-cell exhaustion group, improved overall survival was observed in patients with high neoantigen expression. However, no significant impact of neoantigen expression was found in the T-cell activation group. Such findings suggest that neoantigen expression affect overall survival of breast cancer patients differently given disparate T-cell status. Patients with high level of neoantigen expression survived longer than those with low level of neoantigen expression in ER-positive breast cancer.

In our study, the neoantigens shared by most breast cancer patients (23 out of 729) were *MUC*16/*CA*125. *MUC*16 is overexpressed in breast cancer tumors and associated with disease stages [55]. It increases breast cancer cell proliferation and inhibits tumor necrosis factor-related apoptosis-inducing ligand (TRAIL) [55]. Long-term survivors of pancreatic ductal adenocarcinoma (PDAC) are enriched in *MUC*16/*CA*125 neoantigens, suggesting that *MUC*16 may serve as a candidate immunogenic hotspot in PDAC [56]. Among the long-term survivors, intertumoral and lasting circulating T-cell activity is related to *MUC*16/*CA*125 neoantigens [56]. There were 21 and 19 patients who had neoantigens on *PCDHGC*5 and *PCDHAC*2. *PCDHGC*5 and *PCDHAC*2 belong to protocadherin (PCDH) gene clusters. The family of PCDH genes are downregulated in breast cancer tissues and identified as a new target of aberrant DNA hypermethylation in breast cancer [57].

One limitation of this study is lack of patient specific HLA genotyping. Neoantigens were predicted based on the peptide-HLA binding affinity scores, available on 729 patients from a previous study [20]. Neoantigens were currently predicted on peptides bound to MHC class I molecules only. In the future, we will include MHC class II molecules in the prediction of neoantigen load. Recent studies detected prominent CD4^+^ T-cell responses against immunizing neoantigens in the use of peptide-MHC class I prediction of neoantigens with unknown reasons [32,33,58]. Improved methods that predict the immunogenicity of CD8^+^ antigens and eventually CD4^+^ antigens would further clarify these findings and enhance immunogenicity [32,33,58]. Neoantigens derived from driver mutations in cancer-associated genes will have great potential in immunotherapy. In melanoma, vemurafenib was shown to improve the rates of response and overall survival of patients with the BRAF V600E mutation [59,60,61,62]. Therefore, future investigation on neoantigens that are derived from specific mutations may improve personalized therapies in cancer treatment.

## 5. Conclusions

In this study, we investigated the correlations between neoantigen expression, clinical pathological variables, DNA repair gene expression, and assessed the impact of neoantigen expression and T-cell activation status on patient overall survival in breast cancer. Our results suggest that neoantigen expression varied across hormone receptor status, molecular subtype, and disease stage. Neoantigen expression was associated with the expression levels of 10 DNA repair genes. Moreover, high neoantigen expression was associated with decreased mortality risk in the whole study samples, T-cell exhaustion subgroup, and PR positive subgroup, suggesting that neoantigens can serve as potential immunogenic agents to improve patient survival in breast cancer.

## Figures and Tables

**Table 1:** Characteristics of 729 breast cancer patients.

Variable	N	%
ER	691	
Negative	158	22.9
Positive	533	77.1
PR	689	
Negative	218	31.6
Positive	471	68.4
HER2	498	
Negative	381	76.5
Positive	117	23.5
Molecular subtype	495	
Luminal	393	79.4
Basal-like	71	14.3
HER2-enrich	31	6.3
Stage	709	
I	125	17.6
II	408	57.6
III & IV	176	24.8
Histology	728	
Ductal	604	83.0
Lobular	62	8.5
Mix	44	6.0
Other	18	2.5
Death	729	
No	617	84.6
Yes	112	15.4
	**N**	**Mean (Range)**
Age (mean ± SD ^1^, years)	729	57.7 ± 13.1 (26–90)
Follow-up (months)	729	42.8 (0–282.7)

^1^ SD: standard deviation.

**Table 2:** Relationship between overall survival and neoantigen expression.

Variables	Death	
	HR (95% ${\mathrm{CI}\;}^{1}$	*p*-Value
Neoantigen Expression		
Low	1.00	
High	0.61 (0.38–0.97)	0.038
T-cell Activation		
Exhaustion	1.00	
Activation	0.48 (0.24–0.96)	0.038
Age	1.04 (1.03–1.06)	<0.001
ER		
Negative	1.00	
Positive	0.53 (0.32–0.87)	0.012
Stage		
Stage I	1.00	
Stage II–IV	2.64 (1.39–5.04)	0.003
Histology		
Ductal	1.00	
Lobular	0.73 (0.35–1.51)	0.395
Mix or Other	0.93 (0.48–1.77)	0.817

^1^ CI: confidence interval.

**Table 3:** Association between neoantigen expression and overall survival stratified by T-cell activation status.

Stratification		Death	
Variable	Variables	HR (95% ${\mathrm{CI}\;}^{1}$	*p*-Value
T-cell Exhaustion	Neoantigen Expression		
	Low	1.00	
	High	0.55 (0.34–0.89)	0.016
	Age	1.04 (1.03–1.06)	<0.001
	ER		
	Negative	1.00	
	Positive	0.45 (0.27–0.75)	0.002
	Stage		
	Stage I	1.00	
	Stage II–IV	2.80 (1.38–5.67)	0.004
	Histology		
	Ductal	1.00	
	Lobular	0.58 (0.25–1.30)	0.185
	Mix or Other	0.95 (0.47–1.93)	0.887
T-cell Activation	Neoantigen Expression		
	Low	1.00	
	High	0.76 (0.08–7.44)	0.816
	Age	1.06 (1.00–1.13)	0.049
	ER		
	Negative	1.00	
	Positive	0.89 (0.18–4.34)	0.890
	Stage		
	Stage I	1.00	
	Stage II–IV	5.52 (0.71–42.90)	0.103
	Histology		
	Ductal	1.00	
	Lobular	7.80 (1.00–60.59)	0.050
	Mix or Other	0.48 (0.05–4.44)	0.521

^1^ CI: confidence interval.

**Table 4:** Association between neoantigen expression and overall survival stratified by ER status.

Stratification		Death	
Variable	Variables	HR (95% ${\mathrm{CI}\;}^{1}$	*p*-Value
ER Positive	Neo Expression		
	Low	1.00	
	High	0.61 (0.36–1.04)	0.067
	T-cell Activation		
	Exhaustion	1.00	
	Activation	0.80 (0.34–1.89)	0.613
	Age	1.05 (1.03–1.07)	<0.001
	Stage		
	Stage I	1.00	
	Stage II–IV	2.56 (1.25–5.24)	0.010
	Histology		
	Ductal	1.00	
	Lobular	0.47 (0.19–1.14)	0.096
	Mix or Other	1.01 (0.49–2.07)	0.989
ER Negative	Neo Expression		
	Low	1.00	
	High	0.76 (0.26–2.16)	0.601
	T-cell Activation		
	Exhaustion	1.00	
	Activation	0.32 (0.11–0.99)	0.048
	Age	1.03 (1.00–1.06)	0.061
	Stage		
	Stage I	1.00	
	Stage II–IV	4.93 (0.65–37.26)	0.122
	Histology		
	Ductal	1.00	
	Lobular	4.64 (1.17–18.45)	0.029
	Mix or Other	0.61 (0.13–2.90)	0.538

^1^ CI: confidence interval.

**Table 5:** Association between neoantigen expression and overall survival stratified by PR status.

Stratification		Death	
Variable	Variables	HR (95% ${\mathrm{CI}\;}^{1}$	*p*-Value
PR Positive	Neo Expression		
	Low	1.00	
	High	0.57 (0.32–0.99)	0.046
	T-cell Activation		
	Exhaustion	1.00	
	Activation	0.82 (0.29–2.36)	0.720
	Age	1.05 (1.03–1.07)	<0.001
	ER		
	Negative	1.00	
	Positive	0.85 (0.11–6.38)	0.876
	Stage		
	Stage I	1.00	
	Stage II–IV	2.45 (1.15–5.20)	0.020
	Histology		
	Ductal	1.00	
	Lobular	0.46 (0.19–1.14)	0.095
	Mix or Other	0.97 (0.45–2.10)	0.941
PR Negative	Neo Expression		
	Low	1.00	
	High	0.67 (0.24–1.84)	0.439
	T-cell Activation		
	Exhaustion	1.00	
	Activation	0.45 (0.17–1.16)	0.096
	Age	1.03 (1.00–1.06)	0.036
	ER		
	Negative	1.00	
	Positive	0.63 (0.29–1.38)	0.247
	Stage		
	Stage I	1.00	
	Stage II–IV	3.73 (0.88–15.91)	0.075
	Histology		
	Ductal	1.00	
	Lobular	3.25 (0.92–11.55)	0.068
	Mix or Other	0.77 (0.22–2.69)	0.685

^1^ CI: confidence interval.

## Data Availability

The clinical data is available at The Cancer Genome Atlas (TCGA) breast invasive carcinoma study (http://www.cbioportal.org/ assessed on 1 July 2018). The somatic mutation and normalized RNA sequencing data are available at the Genomic Data Commons (GDC) data portal (https://portal.gdc.cancer.gov/ assessed on 26 September 2019). The predicted neoantigen data is available in a previous study at [doi:10.1016/j.cell.2014.12.033] [20].

## Supplementary Materials

The following are available online at https://www.mdpi.com/article/10.3390/cancers13122879/s1, Table S1: Correlation between neoantigen expression and clinical pathological variables; Table S2: Correlation between neoantigen expression, mutation load, expression of the most shared neoantigens and DNA repair gene expression; Table S3: Relationship between overall survival and expression of the most shared neoantigens; Table S4: Association between expression of the most shared neoantigens and overall survival stratified by ER status; Table S5: Association between expression of the most shared neoantigens and overall survival stratified by PR status; Figure S1: Kaplan–Meier survival curve of expression of the most shared neoantigens; Figure S2: Kaplan–Meier survival curve of expression of the most shared neoantigens stratified by T-cell activation status; Figure S3: Kaplan–Meier survival curve of expression of the most shared neoantigens stratified by ER status; Figure S4: Kaplan–Meier survival curve of expression of the most shared neoantigens stratified by PR status.
