# Supplementary material for: Impact of Neoantigen Expression and T-Cell Activation on Breast Cancer Survival
Source: Cancers (Basel). 2021 Jun 9;13(12):2879. doi: 10.3390/cancers13122879 (PMC8228363; doi:10.3390/cancers13122879)
Supplement: Supplementary file 1 [file cancers-13-02879-s001.zip › cancers-1223396-supplementary.pdf]

Impact of Neoantigen Expression and T-Cell Activation on Breast Cancer Survival

Wenjing Li, Amei Amei, Francis Bui, Saba Norouzifar, Lingeng Lu and Zuoheng Wang

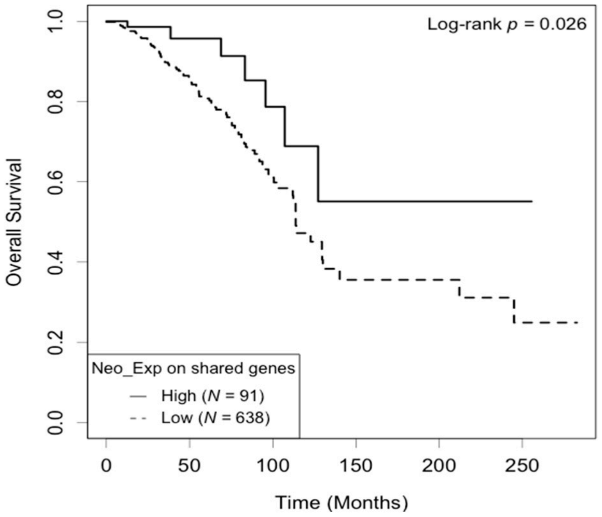

Figure S1. Kaplan-Meier survival curve of expression of the most shared neoantigens. Patients with a high level of neoantigen expression had better overall survival compared to those with a low level ($p = 0.026$).

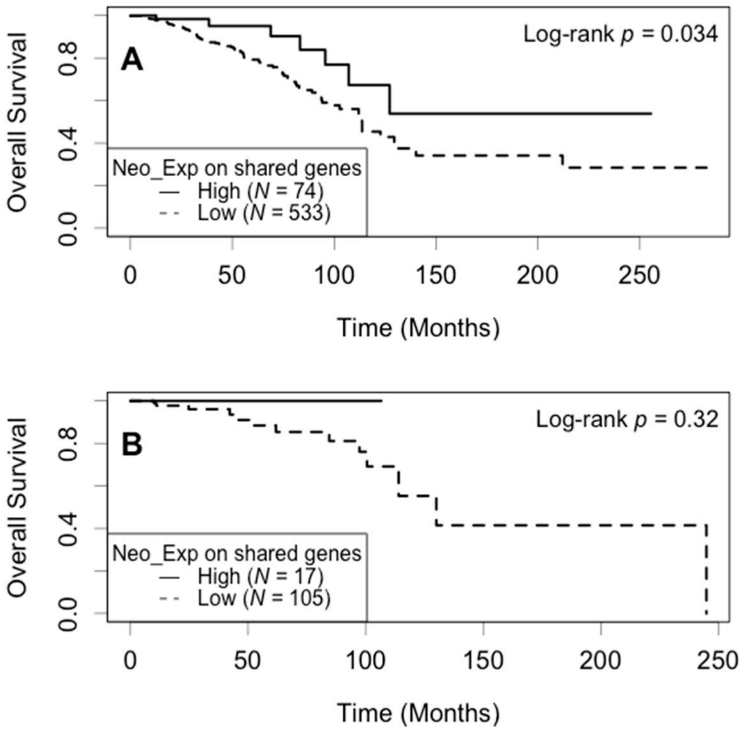

Figure S2. Kaplan-Meier survival curve of expression of the most shared neoantigens stratified by T cell activation status. (A) In the T cell exhaustion subgroup, patients with a high level of neoantigen expression had better overall survival compared to those with a low level ($p = 0.034$); (B) In the T cell activation subgroup, there was no significant difference in survival between patients in the high and low levels of neoantigen expression ($p = 0.32$).

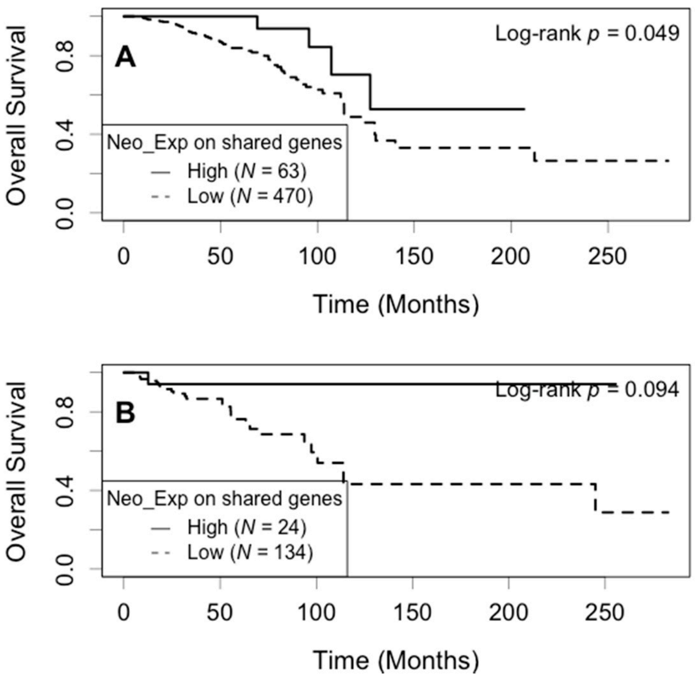

Figure S3. Kaplan-Meier survival curve of expression of the most shared neoantigens stratified by ER status. (A) In the ER positive subgroup, patients with a high level of neoantigen expression had better overall survival compared to those with a low level ($p = 0.049$); (B) In the ER negative subgroup, there was no significant difference in survival between patients in the high and low levels of neoantigen expression ($p = 0.094$).

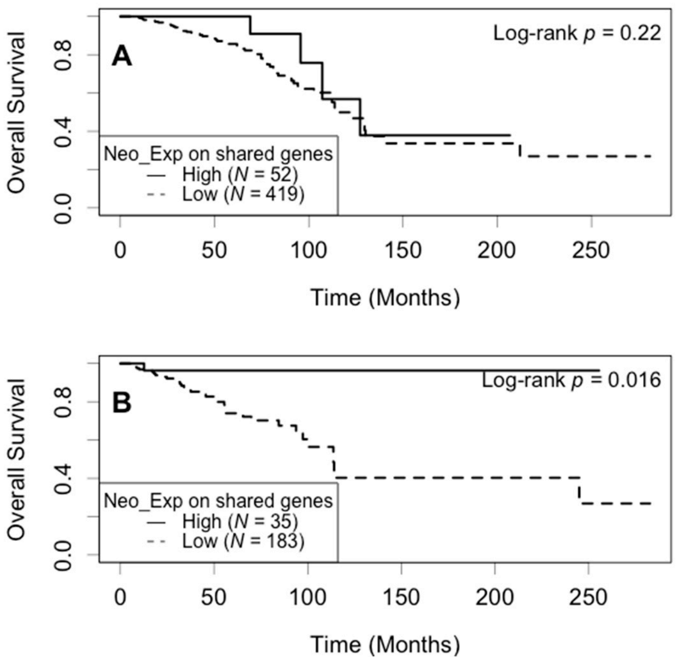

Figure S4. Kaplan-Meier survival curve of expression of the most shared neoantigens stratified by PR status. (A) In the PR positive subgroup, there was no significant difference in survival between patients in the high and low levels of neoantigen expression ($p = 0.22$); (B) In the PR negative subgroup, patients with a high level of neoantigen expression had better overall survival compared to those with a low level ($p = 0.016$).

Table S1. Correlation between neoantigen expression and clinical pathological variables.

Variable	Neoantigen Expression		
	High *N* (%)	Low *N* (%)	*p*-Value
ER			
Positive	405 (76.0)	128 (24.0)	0.016
Negative	135 (85.4)	23 (14.6)	
PR			
Positive	354 (75.2)	117 (24.8)	0.006
Negative	185 (84.9)	33 (15.1)	
HER2			
Positive	101 (86.3)	16 (13.7)	0.073
Negative	298 (78.2)	83 (21.8)	
Molecular subtype			
Luminal	304 (77.4)	89 (22.6)	0.004
Basal-like	64 (90.1)	7 (9.9)	
HER2-enrich	29 (93.5)	2 (6.5)	
Stage			
I	86 (68.8)	39 (31.2)	0.016
II	328 (80.4)	80 (19.6)	
III & IV	142 (80.7)	34 (19.3)	
Histology			
Ductal	476 (78.8)	128 (11.2)	
Lobular	49 (79.0)	13 (21.0)	0.259
Mix	29 (65.9)	15 (34.1)	
Other	14 (77.8)	4 (22.2)	

Table S2. Correlation between neoantigen expression, mutation load, expression of the most shared neoantigens and DNA repair gene expression.

Gene Name	Correlation (*p*-Value)		
	Neoantigen Expression	Mutation Load	Shared Neoantigen Expression
*APEX*1	0.01 (1.000)	0.06 (1.000)	−0.02 (1.000)
ATM	−0.07 (1.000)	−0.24 (7.03×10^{-10})	−0.06 (1.000)
BLM	0.26 (5.02×10^{-11})	0.36 ($<5.28 \times 10^{-15}$)	0.12 (0.026)
*BRCA*1	0.06 (1.000)	0.04 (1.000)	0.03 (1.000)
*BRCA*2	0.16 (3.45×10^{-4})	0.21 (4.98×10^{-7})	0.01 (1.000)
*CHEK*1	0.20 (9.98×10^{-7})	0.32 ($<5.28 \times 10^{-15}$)	0.08 (0.542)
*CHEK*2	0.24 (1.68×10^{-9})	0.33 ($<5.28 \times 10^{-15}$)	0.12 (0.024)
*ERCC*1	−0.14 (4.95×10^{-3})	−0.16 (1.99×10^{-4})	−0.08 (0.600)
*ERCC*4	0.04 (1.000)	−0.08 (0.775)	0.01 (1.000)
*ERCC*6	−0.02 (1.000)	−0.08 (0.614)	−0.02 (1.000)
FANCG	0.06 (1.000)	0.20 (1.67×10^{-6})	0.06 (1.000)
*MLH*1	−0.01 (1.000)	−0.14 (2.28×10^{-3})	−0.01 (1.000)
*MSH*2	0.17 (9.83×10^{-5})	0.22 (2.79×10^{-8})	0.05 (1.000)
*MSH*3	−0.10 (0.240)	−0.24 (6.66×10^{-10})	−0.06 (1.000)
MUTYH	0.03 (1.000)	0.10 (0.159)	0.00 (1.000)
*OGG*1	0.00 (1.000)	−0.05 (1.000)	0.02 (1.000)
*RAD*51	0.23 (9.46×10^{-9})	0.36 ($<5.28 \times 10^{-15}$)	0.09 (0.498)
*RPAP*1	−0.05 (1.000)	−0.12 (0.037)	−0.01 (1.000)
XPA	−0.18 (1.19×10^{-5})	−0.32 ($<5.28 \times 10^{-15}$)	−0.06 (1.000)
XPC	−0.17 (7.58×10^{-5})	−0.35 ($<5.28 \times 10^{-15}$)	−0.12 (0.025)
*XRCC*1	−0.08 (0.840)	−0.10 (0.241)	−0.07 (1.000)
*XRCC*2	0.19 (5.18×10^{-6})	0.30 (5.33×10^{-15})	0.11 (0.071)
*XRCC*3	0.08 (0.998)	0.16 (5.92×10^{-4})	0.03 (1.000)

XRCC4	0.02 (1.000)	−0.06 (1.000)	−0.05 (1.000)

Table S3. Relationship between overall survival and expression of the most shared neoantigens.

Variables	Death	
	HR (95% CI[1])	p−Value
Most Shared Neoantigen Expression		
Low	1.00	
High	0.30 (0.11−0.82)	0.019
T Cell Activation		
Exhaustion	1.00	
Activation	0.46 (0.23−0.92)	0.029
Age	1.04 (1.02−1.06)	<0.001
ER		
Negative	1.00	
Positive	0.58 (0.36−0.94)	0.028
Stage		
Stage I	1.00	
Stage II−IV	2.78 (1.46−5.31)	0.002
Histology		
Ductal	1.00	
Lobular	0.81 (0.40−1.66)	0.568
Mix or Other	1.01 (0.53−1.94)	0.971

Table S4. Association between expression of the most shared neoantigens and overall survival stratified by ER status.

Stratification Variable	Variables	Death	
		HR (95% CI[1])	p−value
ER Positive	Neo Expression		
	Low	1.00	
	High	0.28 (0.09−0.90)	0.033
	T Cell Activation		
	Exhaustion	1.00	
	Activation	0.73 (0.31−1.73)	0.477
	Age	1.05 (1.03−1.07)	<0.001
	Stage		
	Stage I	1.00	
	Stage II−IV	2.78 (1.36−5.69)	0.005
	Histology		
	Ductal	1.00	
	Lobular	0.53 (0.22−1.26)	0.153
	Mix or Other	1.15 (0.56−2.37)	0.703
ER Negative	Neo Expression		
	Low	1.00	
	High	0.43 (0.06−3.29)	0.413
	T Cell Activation		
	Exhaustion	1.00	
	Activation	0.32 (0.11−0.99)	0.049
	Age	1.03 (1.00−1.06)	0.083
	Stage		
	Stage I	1.00	
	Stage II−IV	4.73 (0.62−35.95)	0.132
	Histology		
	Ductal	1.00	
	Lobular	4.73 (1.27−17.68)	0.021
	Mix or Other	0.66 (0.14−3.11)	0.603

[1] CI: confidence interval.

Table S5. Association between expression of the most shared neoantigens and overall survival stratified by PR status.

Stratification Variable	Variables	Death HR (95% CI[1])	*p*–Value
PR Positive	Neo Expression		
	Low	1.00	
	High	0.37 (0.11–1.21)	0.100
	T Cell Activation		
	Exhaustion	1.00	
	Activation	0.70 (0.24–1.98)	0.497
	Age	1.05 (1.03–1.07)	<0.001
	ER		
	Negative	1.00	
	Positive	0.80 (0.11–5.97)	0.826
	Stage		
	Stage I	1.00	
	Stage II–IV	2.64 (1.24–5.63)	0.012
	Histology		
	Ductal	1.00	
	Lobular	0.55 (0.23–1.32)	0.179
	Mix or Other	1.14 (0.53–2.48)	0.734
PR Negative	Neo Expression		
	Low	1.00	
	High	0.17 (0.02–1.25)	0.082
	T Cell Activation		
	Exhaustion	1.00	
	Activation	0.45 (0.17–1.16)	0.100
	Age	1.02 (1.00–1.05)	0.083
	ER		
	Negative	1.00	
	Positive	0.75 (0.34–1.66)	0.485
	Stage		
	Stage I	1.00	
	Stage II–IV	3.68 (0.87–15.60)	0.078
	Histology		
	Ductal	1.00	
	Lobular	3.50 (1.02–12.03)	0.047
	Mix or Other	0.81 (0.23–2.82)	0.739

[1] CI: confidence interval.
